# Alginate films augmented with chlorhexidine hexametaphosphate particles provide sustained antimicrobial properties for application in wound care

**DOI:** 10.1007/s10856-020-06370-0

**Published:** 2020-03-11

**Authors:** Peter F. Duckworth, Sarah E. Maddocks, Sameer S. Rahatekar, Michele E. Barbour

**Affiliations:** 1 Oral Nanoscience, Bristol Dental School, University of Bristol, Bristol, UK; 2 ACCIS, Queens School of Engineering, University of Bristol, Bristol, UK; 3 Cardiff School of Health Sciences, Cardiff Metropolitan University, Cardiff, UK; 4 School of Aerospace, Transport and Manufacturing, University of Cranfield, Bedford, UK; 5 Pertinax Pharma Ltd, Bristol, UK

## Abstract

All chronic wounds are colonised by bacteria; for some, colonisation progresses to become infection. Alginate wound dressings are used for highly exuding chronic wounds as they are very absorbent, taking up large quantities of exudate while maintaining a moist wound bed to support healing. Some alginate dressings are doped with antimicrobials, most commonly silver, but evidence regarding the efficacy of these is largely inconclusive. This manuscript describes the development and in vitro assessment of alginate materials doped with chlorhexidine hexametaphosphate (CHX-HMP), a sparingly soluble salt which when exposed to aqueous environments provides sustained release of the common antiseptic chlorhexidine. Comparator materials were a commercial silver alginate dressing material and an alginate doped with chlorhexidine digluconate (CHXdg). CHX-HMP alginates provided a dose-dependent CHX release which was sustained for over 14 days, whereas CHXdg alginates released limited CHX and this ceased within 24 h. CHX-HMP and silver alginates were efficacious against 5 major wound pathogens (MRSA, *E. coli, P. aeruginosa, K. pneumoniae, A. baumannii*) in a total viable count (TVC) and an agar diffusion zone of inhibition (ZOI) model. At baseline the silver alginate was more effective than the CHX-HMP alginate in the TVC assay but the CHX-HMP alginate was the more effective in the ZOI assay. After 7 days’ artificial aging the CHX-HMP alginate was more effective than the silver alginate for four of the five bacteria tested in both assays. These materials may ultimately find application in the development of wound dressings for chronic wounds that provide sustained antimicrobial protection.

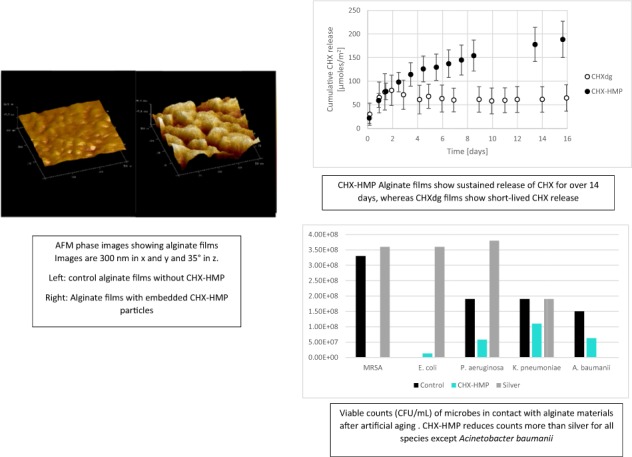

## Introduction

Chronic wounds present substantial challenges to healthcare practitioners and are a source of discomfort and debility to millions of people worldwide. Among the most common chronic wounds are diabetic foot ulcers and venous leg ulcers, both of which are becoming more numerous as the population ages. Since 1980, the number of adults with type 2 diabetes has almost quadrupled, with lower and middle income countries the most affected [1]; 25% of these people will develop a diabetic foot ulcer in their lifetime, and 50% of those will become infected [2]. Venous ulcers are the most common chronic leg wounds, and prevalence rises with age, affecting 1.7% of people over 65 [3]. All of these chronic wounds can be considered to be colonised by bacteria; only some progress to clinical infection, and the factors which determine whether and when this occurs are not well understood, resulting in uncertainty as to how to best prevent and treat them [4].

Alginate-based dressings are used to dress many chronic wounds, including diabetic foot and venous leg ulcers. Alginate dressings are highly absorbent, absorbing 15–20× their weight in fluid; wound exudate is taken up by the dressing which forms into a gel, preventing leakage and maintaining a moist environment to support healing [5]. Antimicrobial alginate dressings are available, most commonly containing silver as the antimicrobial agent. While there is some evidence that silver-impregnated dressings, including but not limited to alginates, can improve short-term healing of leg wounds, there is insufficient evidence to conclude whether these improve healing long-term [6]. There is also insufficient evidence to conclude whether they aid healing of diabetic foot ulcers [7], and a Cochrane review of the efficacy of silver to prevent wound infection found low quality evidence to suggest that silver products can *detrimentally* affect wound healing [8]. There is in vitro evidence to indicate that the antimicrobial efficacy of silver alginate dressings diminishes over time, with an agar diffusion assay revealing a reduction in zone of inhibition of 60% after 1 day and no zone at all at 7 days [9].

Chlorhexidine (CHX) is a broad-spectrum antiseptic used widely in health care. In wound care it is most commonly used as a pre-operative skin disinfectant, in the form of a solution of CHX digluconate, and in this use it is accepted to be effective in reducing the microbial population on the skin [10]. It is also used as a component of some dressings, although less commonly than silver, and not in conjunction with alginate, at least in a commercial product. The most commonly encountered dressing making use of CHX are securement devices for catheters for vascular access; these are usually composed of CHX-impregnated polyurethane foam or polyurethane-coated gel, and have been found to reduce catheter microbial colonisation and consequent catheter-related blood stream infection [11].

Commercial CHX products including the vascular access devices described above employ CHX digluconate, which is readily soluble in water. This has limited its use in dressings, particularly those which are required to absorb a large quantity of exudate, as the solubility of the CHX salt means that it is released rapidly on contact with wound fluid, resulting in a high initial concentration and no sustained release. Recently, condensed phosphate salts of CHX have been reported [12]. CHX hexametaphosphate (CHX-HMP) is a sparingly soluble CHX salt which, when in contact with aqueous media, effects a slow release of soluble, active, CHX over an extended period, the duration of which and the resultant concentration of CHX in the local environment being dependent on factors such as fluid flow. The aim of this study was to explore whether alginate doped with CHX-HMP could have sustained and effective antimicrobial properties that may support its ultimate use in absorbent dressings for chronic wounds.

## Materials and methods

Other than where stated, all chemicals and reagents were supplied by Sigma-Aldrich (Gillingham, UK).

### Synthesis and analysis of CHX-HMP

Equal volumes of CHX digluconate (CHXdg) (10 mM) and sodium hexametaphosphate (10 mM) were rapidly and simultaneously poured into a beaker being rapidly stirred. The resulting suspension of CHX-HMP particles was used on the same day and ultrasonicated (Grant MXB6 ultrasonicating water bath, Grant Instruments, Cambridge, UK) for 15 min immediately prior to use. To obtain pure CHX-HMP for chemical analysis the suspension was cooled in ice and Büchner filtered, the filtrate washed with ice cooled water and obtained as a white amorphous solid. Ultraviolet (UV) spectrophotometry of CHX-HMP suspensions and CHXdg solutions was performed using a Lambda 35 spectrophotometer (Perkin Elmer, Massachusetts, USA). FT-IR spectra of CHX-HMP precipitate were recorded in the range 650–4000 cm^−1^ using a Spectrum 100 (Perkin Elmer, Massachusetts, USA) and peaks reported as νmax (neat)/cm^−1^ which refers to νmax in wavenumbers. Elemental analysis for C, H, N and P was carried out by the University of Bristol microanalytical laboratory. Other properties of the CHX-HMP have been reported in earlier publications [12–14].

### Preparation and characterisation of alginate films containing CHX-HMP or CHXdg

PROTANAL LF10/60FT (FMC Health and Nutrition, Philadelphia, USA), a ‘high G’ alginate with a G:M ratio of 60–70:30–40, was used. A 2 wt% aqueous solution of alginate was prepared by adding dry alginate to a rapidly stirring aqueous suspension of CHX-HMP equivalent to 6, 3, 1, 0.5, 0.1, 0.05 and 0.01 wt% (for comparison: [CHX] = 4.88 mM for 6 wt%). Alginate films prepared without any CHX are denoted Alg-ctrl. Specimens are denoted as X-CHX-HMP where X refers to the wt% CHX-HMP in the alginate; for example, 6-CHX-HMP dressings contain 6 wt% CHX-HMP. 17.5 g of the suspensions were poured into petri dishes (ø = 90 mm) and the water allowed to evaporate at room temperature for 3 days. CaCl_2_ (30 mL, 0.18 M, 2 wt%) was added to the petri dishes for 25 min to permit cross-linking. The cross-linked alginate films, which were ~1 mm thick, were then removed from the petri dishes, washed with distilled water and air dried on parafilm. CHXdg alginate films were prepared as for CHX-HMP films by dissolving the alginate in CHXdg solution diluted to the required concentration, instead of the CHX-HMP suspension, and these are referred to as X-CHXdg where X again represents the concentration of CHX-HMP that provides the equivalent concentration of CHX in the material. Disk shaped specimens (ø = 10 mm) were cut from theses material and used for subsequent work. 6-CHX-HMP and Alg-ctrl films were analysed using atomic force microscopy (AFM; Nanoscope IIIa, Veeco, New York, USA) in tapping mode with topography and phase imaging.

Tegaderm Alginate Ag^TM^ (3M, Minnesota, USA; abbreviated to ‘Ag-Alg’), a commercially available non-woven fibrous alginate mat containing silver sodium hydrogen zirconium phosphate as an active antibacterial agent, was used as a positive control. Discs (ø = 10 mm) were cut from this material under sterile conditions and these used for subsequent work.

In some instances, specimens of 6-CHX-HMP and Ag-Alg were investigated after a period of aging to assess the properties of the material after a period of use. To age specimens, discs were sealed in air-tight sterile Kilner^TM^ jars containing distilled water (2.25 mL per disk). Jars remained stationary for seven days with 4× inversion on day 1, 3 and 7. Specimens were then removed, rinsed, immersed in water (1 min), rinsed, air dried and used.

### Chlorhexidine elution from dressings

CHX elution was assessed using UV spectroscopy at 254 nm with CHX concentration derived by comparison to CHXdg standards at 5–50 µM CHX. Alginate discs with 3- and 6-CHX-HMP and 6-CHXdg were placed in individual semi-micro cuvettes (BRAND^®^ semi-micro cuvettes, BRAND GMBH + CO KG, Wertheim, Germany), covered with distilled water (2.25 mL) and the cuvette sealed with a lid and parafilm (*n* = 30 for each CHX doping). Cuvettes were agitated throughout using a New Brunswick Scientific G-25 incubator shaker at 150 rpm and 37 °C (Eppendorf, Hamburg, Germany) and the absorbance (254 nm) was recorded frequently over a two-week period.

### Microbial growth inhibition assays

Microbiological assays were conducted using: Methicillin-resistant *Staphylococcus aureus* EMRSA-15, *Pseudomonas aeruginosa* ATCC9027, *Escherichia coli* NCTC10418, *Klebsiella pneumoniae* ATCC10031 and *Acinetobacter baumannii* 121J6. Bacteria suspensions were equilibrated to a 0.5 MacFarland standard which is ~1.5 × 10^8^ colony forming units (CFU)/mL at A600. All bacteria were cultured aerobically at 37 °C in nutrient broth.

To establish total viable counts (TVC) of bacteria incubated with the alginate materials, single 10 mm disk specimens of Alg-ctrl, 3-CHX-HMP, 6-CHX-HMP and Ag-Alg were placed in sterile, 5 mL plastic bijou containers containing 1 mL nutrient broth and inoculated with equilibrated bacterial suspension (10 μL). Each bacterium was tested separately, and nine repeats of each material-bacteria combination were assessed. The culture from each vial was discarded following incubation (24 h, 37 °C), and each specimen transferred into a fresh container containing 1 mL phosphate-buffered saline (PBS). This was vortexed (2200 rpm, 20 s) dislodging any bacteria contained within the specimens into the PBS. Serial dilutions (10^−1^–10^−12^) were prepared using PBS, these were enumerated using the TVC method [15].

To assess zones of inhibition (ZOI) in an agar diffusion model, bacterial lawns (100 μL) were prepared on a nutrient agar plate (ø = 90 mm) using the spread plate method and allowed to air-dry before specimens were placed onto the lawns. These were incubated (24 h, 37 °C) and any ZOI measured using digital callipers. Each bacterium was tested separately; nine repeats of each material-bacteria combination were assessed.

## Results

### Characterisation of CHX-HMP particles and alginate films

**Fig. 1:**
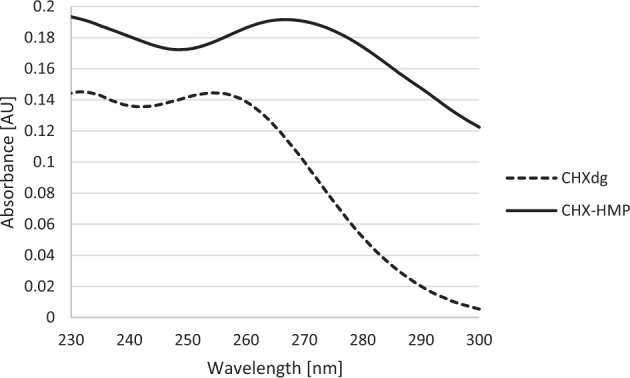
UV absorption spectra of CHXdg solution and CHX-HMP suspension. There is a bathochromic shift in the first absorption maximum following the precipitation reaction, from 254 nm for CHXdg to 268 nm for CHX-HMP

A bathochromic shift in the UV absorption maximum of CHX accompanies the reaction between CHX and HMP, from 254 to 268 nm (Fig. 1) [16], suggesting an increase in delocalisation within the molecular structure. This indicates that an electrostatic interaction between the polar groups of each individually soluble ion—biguanides and phosphates—is involved in the reaction.

**Fig. 2:**
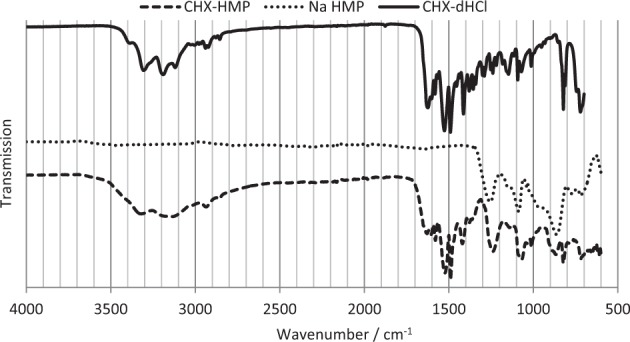
FT-IR spectra of CHX-HMP, sodium hexametaphosphate (Na HMP) and chlorhexidine dihydrochloride (CHX-dHCl)

Using Büchner filtration, CHX-HMP was obtained as a white amorphous solid. Elemental analysis is shown in Table 1, and compared with predicted values using a 3:1 CHX:HMP ratio, i.e. [C_22_H_28_Cl_2_N_10_]_3_[O_18_P_6_]. FTIR spectra are shown in Fig. 2 and indicated νmax (neat): 3321 (NH), 3150 (C–H_chlorophenyl_), 2936 & 2860 (C–H_methanediyls_), 1628 (ArNHC(= N-H)NHAr) (aromatic guanidine absorptions), 1604 ((CH_3_)_2_NC(= N–H)C(CH_3_)_2_) (aliphatic guanidine absorptions), 1580 & 1524 (C = N & C = C_aromatic_), 1490 (C–H_methanediyl_), 1420 (C = N & C = C_aromatic_), 1239 (*P* = O), 1071 (P–O), 1012 (C = N & C = C_aromatic_), 867 (P–O–P), 824 (C–H_aromatic_), 729 (C–Cl_aromatic_) cm^−1^ [17–23].

**Table 1:** Elemental analysis of CHX-HMP precipitate

Element	Recorded	Predicted using a 3:1 CHX:HMP ratio
C	40.1	39.8
H	5.3	4.3
N	20.0	21.1
P	8.6	9.3

**Fig. 3:**
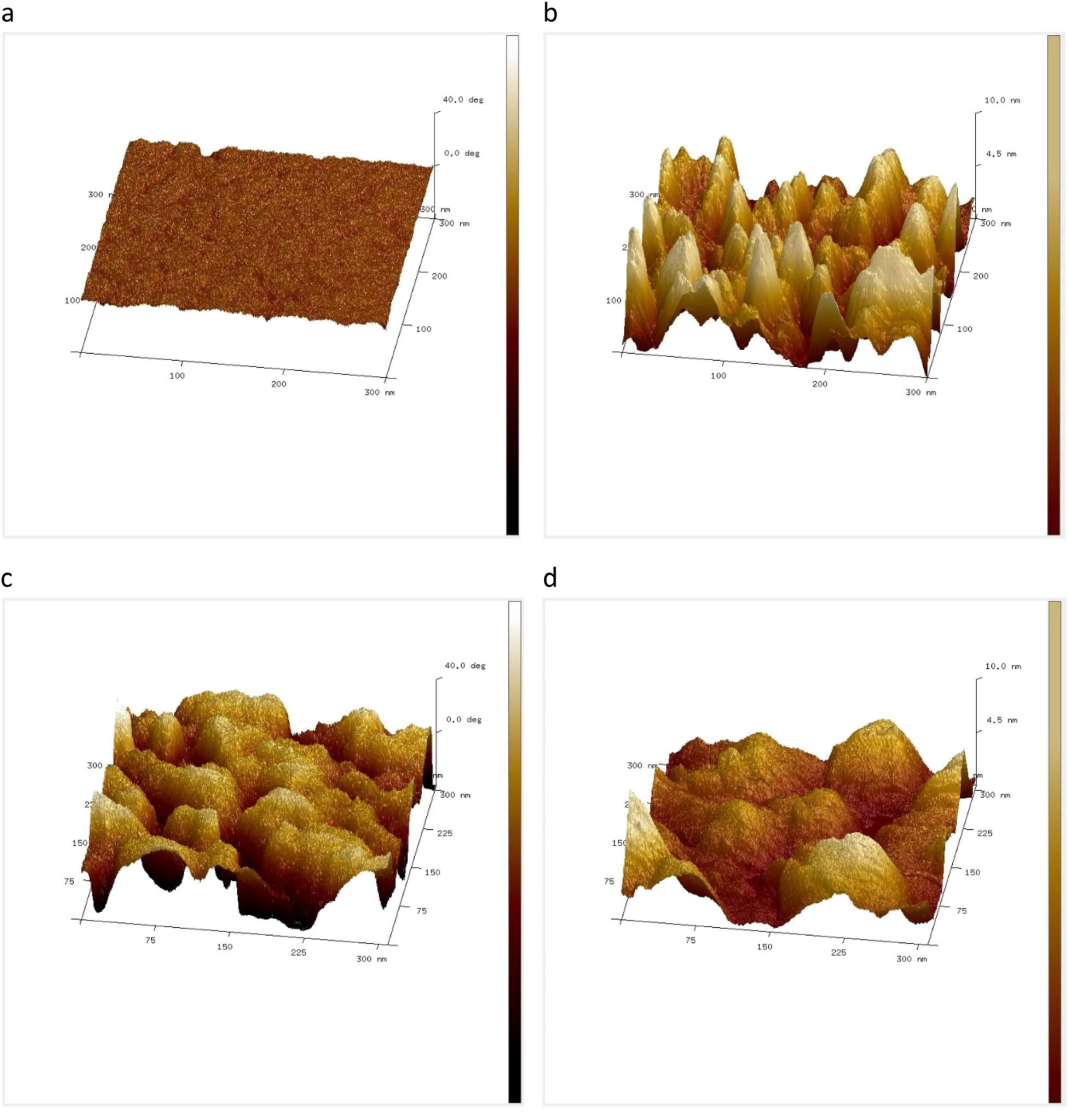
AFM images of alginate films. **a** phase image and **b** topography image of control film Alg-ctrl; **c** phase image and **d** topography image of 6-CHX-HMP films. The x and y axes are 300 nm in each case. The z axis represents 40 degrees in the case of phase (**a** and **c**) and 10 nm in the case of topography (**b** and **d**). Both Alg-ctrl and 6-CHX-HMP films are rough in topography, but the Alg-ctrl film shows little phase variation compared with the 6-CHX-HMP film, indicating a variability in stiffness of the 6-CHX-HMP film due to the presence of embedded hard, rigid CHX-HMP particles within the soft, flexible alginate film

AFM topography images indicated a rough surface texture for both Alg-ctrl and those containing CHX-HMP, but phase imaging revealed evidence of variation in stiffness and subsurface heterogeneity in the CHX-HMP-impregnated alginate which were not observed in the control alginate Alg-ctrl (Fig. 3).

### Chlorhexidine elution from dressings

**Fig. 4:**
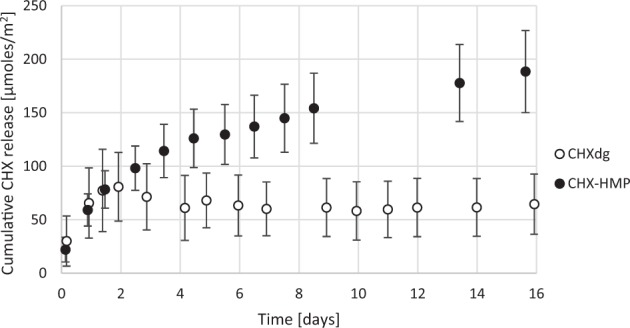
Cumulative CHX release from 6-CHX-HMP and CHXdg alginate films. Error bars represent standard deviations

Elution of CHX from 6-CHX-HMP and 6-CHXdg alginate films are shown in Fig. 4. Elution of CHX from the CHXdg film ceased at ~22 h whereas for the CHX-HMP film CHX release was more sustained and was still ongoing at 16 days. A linear dose response was observed; the average elution of CHX from alginate with 3-CHX-HMP loading over the period was 50% of that of 6-CHX-HMP loading (data not shown).

### Microbial growth inhibition assays

TVCs after 24 h immersion with specimens as prepared and after 7 days’ aging are shown in Table 2, and ZOI in Table 3. The CHX-HMP and commercial silver alginate materials all showed significant antimicrobial efficacy in the TVC and agar diffusion models. The CHX-HMP showed a dose response, with alginates containing more CHX-HMP effecting a greater reduction in TVCs and a larger ZOI. The ZOI of a wide range of CHX-HMP loadings was investigated to establish the minimum CHX-HMP that still showed a ZOI; this varied from 0.05 wt% to 1 wt% depending on the species of microbe. The 3-CHX-HMP and 6-CHX-HMP alginates showed greater ZOI with all microbes except *A. baumannii*, but less reduction in TVC, than the silver alginate. When the silver and 6-CHX-HMP alginates were subjected to 7 days’ aging in distilled water, the CHX-HMP alginates showed greater ZOI than the silver alginate and a greater reduction in TVCs, since aged silver alginates showed no reduction in TVCs whereas CHX-HMP alginates reduced TVCs by at least a factor of 10 except for *P. aeruginosa*.

**Table 2:** Total viable counts of 5 microbes after incubation with CHX-HMP alginates and a commercial silver alginate, with and without aging, and the reduction to the nearest factor of 10 with respect to the control alginate which contained no antimicrobial

	Alg-ctrl		3-CHX-HMP		6-CHX-HMP alginate				Ag-Alg			
	Before aging	After aging	Before aging		Before aging		After aging		Before aging		After aging	
	CFU/mL	CFU/mL	CFU/mL	Reduction	CFU/mL	Reduction	CFU/mL	Reduction	CFU/mL	Reduction	CFU/mL	Reduction
MRSA	3.6 × 10^9^	3.3 × 10^8^	1.8 × 10^5^	10^4^	6.8 × 10^4^	10^5^	1.6 × 10^5^	10^3^	0	10^9^	3.6 × 10^8^	NR
*E. coli*	5.0 × 10^9^	4.7 × 10^8^	5.3 × 10^5^	10^4^	5.4 × 10^4^	10^5^	1.3 × 10^7^	10^1^	0	10^9^	3.6 × 10^8^	NR
*P. aeruginosa*	6.8 × 10^7^	1.9 × 10^8^	7 × 10^6^	10^1^	8.0 × 10^4^	10^3^	5.8 × 10^7^	10^1^	0	10^7^	3.8 × 10^8^	NR
*K. pneumoniae*	2.1 × 10^8^	1.9 × 10^8^	1.8 × 10^7^	10^1^	1.7 × 10^5^	10^3^	1.1 × 10^8^	NR	0	10^8^	1.9 × 10^8^	NR
*A. baumannii*	3.2 × 10^8^	1.5 × 10^8^	4.5 × 10^7^	10^1^	1.0 × 10^7^	10^1^	6.3 × 10^7^	10^1^	0	10^8^	7.2 × 10^5^	10^3^

3-CHX-HMP specimens were not investigated after aging. The silver alginates show total kill at baseline but no antimicrobial efficacy after aging, with the exception of A. baumannii. The experimental CHX-HMP alginates show a dose response, with the 6-CHX-HMP effecting a greater reduction in CFU than 3-CHX-HMP, and show a sustained efficacy, with a reduction in CFU after aging, although this was less than the reduction in CFU without aging

*NR* no reduction

**Table 3:** Zones of inhibition in mm around 10 mm disk-shaped specimens of experimental alginates with a range of CHX-HMP loadings and a commercial silver alginate

	Alg-ctrl	0.01-CHX-HMP	0.05-CHX-HMP	0.1-CHX-HMP	0.5-CHX-HMP	1-CHX-HMP	3-CHX-HMP	6-CHX-HMP	Ag-Alg	AGED 6-CHX-HMP	AGED Ag-Alg
MRSA	NZ	NZ	12.1	13.9	16.6	19.6	21.0	22.6	15.5	18.0	11.7
*E. coli*	NZ	NZ	10.3	11.2	13.1	14.7	18.4	19.7	12.9	16.9	12.5
*P. aeruginosa*	NZ	NZ	NZ	NZ	10.5	10.6	13.5	14.2	12.1	14.1	12.5
*K. pneumoniae*	NZ	NZ	NZ	NZ	10.8	12.7	15.6	16.5	11.9	14.1	11.9
*A. baumannii*	NZ	NZ	NZ	NZ	NZ	10.7	13.0	14.9	16.1	12.3	13.3

The 6-CHX-HMP alginate and Ag-Alg were also investigated after 7 days’ aging. NZ indicates there was no zone; there were in some cases some limited inhibition of growth under the disk

## Discussion

Alginate is a biocompatible polysaccharide used widely in wound dressing products due to its ability to absorb exudate and establish a moist environment supportive of healing whilst retaining its mechanical integrity [24]. Antimicrobials and other drugs embedded within the alginate can help to manage bioburden within the dressing and may also be used to deliver drug directly to the wound bed. The aim of incorporating CHX-HMP into an alginate matrix was to establish whether alginate was a suitable matrix for the use of CHX-HMP in antimicrobial wound dressings. CHX is widely used in wound care but more commonly as a skin disinfectant and in intravenous securement devices rather than in dressings for chronic wounds. However, clinicians are familiar with the broad-spectrum efficacy of CHX and its long history of use and as such a sustained release CHX wound dressing might find favour among medical professionals supporting patients with chronic wounds. The polyphosphate counterion is benign, used as a food additive and in oral care products such as toothpastes. The mechanism of CHX release from CHX-HMP is understood to be a gradual dissolution of the CHX-HMP material followed by diffusion of the aqueous CHX ion.

CHX-HMP was precipitated instantaneously upon mixing of aqueous solutions of CHX and HMP ions (Equation 1). The composition of the salt was found to be 3 CHX ions to 1 HMP ion (Table 1) with FT-IR showing the absence of gluconate in the isolated CHX-HMP (Fig. 2).

$$\begin{array}{*{20}{c}} {3\left( {\left[ {{\mathrm{Chlorhexidine}}} \right]^{2 + }\left[ {{\mathrm{Gluconate}}} \right]_2^ - } \right) + \left( {\left[ {{\mathrm{Sodium}}} \right]_6^ + \left[ {{\mathrm{Hexamaetaphosphate}}} \right]^{6 - }} \right)} \\ \downarrow \\ {\left( {\left[ {{\mathrm{Chlorhexidine}}} \right]_3^{2 + }\left[ {{\mathrm{Hexamaetaphosphate}}} \right]^{6 - }} \right) \downarrow +\; 6\left( {\left[ {{\mathrm{Sodium}}} \right]^ + \left[ {{\mathrm{Gluconate}}} \right]^ - } \right)} \end{array}$$

Equation 1. Precipitation of chlorhexidine hexametaphosphate from solutions of chlorhexidine digluconate and sodium hexametaphosphate.

Alginate films were prepared with a range of dopings of CHX-HMP particles, and AFM phase mode images of 6-CHX-HMP films reveal the presence of stiff particulate inclusions whereas topography images were broadly indistinguishable from control alginate films (Fig. 3), which is consistent with solid particles of CHX-HMP being embedded within the alginate film rather than on the surface. This is consistent with previous findings, in that CHX-HMP exhibits low solubility and a protracted equilibration in water [12], and as such it is highly unlikely that the CHX-HMP particles would dissolve during the process of preparing the alginate film. The opaque appearance of the CHX-HMP films compared with comparatively translucent control films provides further evidence that there were intact CHX-HMP particles present within the alginate.

Films containing CHXdg were prepared using a solution of CHXdg containing the same wt% of CHX as the 6-CHX-HMP suspension, to compare the kinetics of the soluble CHX cation release from films prepared with CHX-HMP and CHXdg. CHX was released rapidly from films containing the highly soluble CHXdg, reaching completion in under 24 h, whereas for the CHX-HMP films the CHX release continued for over 14 days, and was still ongoing at the conclusion of the measurements (Fig. 4), illustrating that the alginate matrix supported the free diffusion of solubilised CHX out of the alginate and into the surrounding environment, potentially aided by the presence of HMP which prevented the CHX from interacting with the alginate directly. Although the CHXdg films were prepared with the same total concentration of CHX as the CHX-HMP (allowing for both soluble and bound CHX), the total release of CHX from the CHXdg films was lower than that for CHX-HMP films. This might indicate that a proportion of the CHX from CHXdg was either not retained in the alginate or was removed during the cross-linking or brief rinsing periods required for preparation. Alternatively it might be that the CHX cation from CHXdg interacted and bound with the alginate itself, thus was not able to leach out of the film in the same way as the HMP-shielded CHX.

The CHX-HMP alginate films showed a dose-dependent antimicrobial efficacy against laboratory strains of five common wound infecting pathogens. *S. aureus, P. aeruginosa* and *E. coli* were selected on the basis that they are some of the most common pathogens isolated from chronic wounds [25–27]; a methicillin-resistant strain of *S. aureus* was utilised in this study on the basis of its importance in the hospital and community settings where chronic wounds are most commonly managed, and with respect to the observation that some MRSA strains exhibit reduced susceptibility to CHX [28], thus providing a more robust challenge to the novel CHX technology in this study. *K. pneumoniae* and *A. baumannii* have historically been less associated with chronic wounds, but these frequently multi-drug-resistant species are increasingly believed to play an important role in wound infections, particularly in wounds sustained by military personnel in conflict zones [29, 30] and in burn wound infections [31, 32].

The microbial counting assessment conducted on bacteria incubated with alginate films indicated that CHX-HMP and Ag-Alg both reduced TVCs. When alginate films were used as prepared/supplied, the Ag-Alg effected a greater reduction in TVCs; no bacteria could be retrieved indicating a complete eradication of each species (10^9^ reduction compared to the control alginate), whereas with 6-CHX-HMP the reduction ranged from 10^1^ (*A. baumannii*) to 10^5^ (MRSA, *E. coli*) and with 3-CHX-HMP the reduction ranged from 10^1^ (*A. baumannii, K. pneumoniae, P. aeruginosa*) to 10^4^ (MRSA, *E. coli*). However, after a 7 day period of aging the 6-CHX-HMP alginate retained some efficacy, reducing TVCs for four of the five bacteria with a reduction of 10^1^ (*E. coli, P. aeruginosa* and *A. baumannii*) to 10^3^ (MRSA), failing only to reduce counts of *K. pneumoniae*. Given that *K. pneumoniae* produces a mucoid layer that renders the species particularly difficult to eradicate using antimicrobials, it is not surprising that this presented a greater challenge to the technology. The silver alginate, however, only reduced TVCs of *A. baumannii* (10^3^) and did not reduce counts of the other species.

The agar diffusion test findings illustrated a dose- and species-dependent efficacy of the CHX-HMP doped alginates. For some bacteria– MRSA, *E. coli* – a doping of as low as 0.05 wt% was sufficient to prevent microbial growth, whereas the threshold concentration was greater with the other bacteriatested; 0.5 wt% for *P. aeruginosa* and *K. pneumoniae* and 1 wt% for *A. baumannii*. For all bacteria, the greater the CHX-HMP concentration (above the threshold noted above), the larger the ZOI. The CHX-HMP alginates provided broadly comparable ZOI to the commercial silver alginate, although ZOI with CHX-HMP were slightly larger than with silver for MRSA, *E. coli, P. aeruginosa* and *K. pneumoniae* but slightly smaller with *A. baumannii*. After the alginates were aged for 7 days, the agar diffusion test showed broadly similar results, with both 6-CHX-HMP and commercial silver alginates showing a slight reduction in ZOI but a ZOI was nevertheless still observed with all bacteria tested.

The 7 day aging protocol was adopted in order to allow any readily soluble, motile antimicrobial to be released, in analogy to the process of wound exudate building up in a dressing, which would usually remain in place on the wound for 7 days. The CHX-HMP technology is by design a controlled release material and as such it is important to consider whether its properties could support a product which retains its efficacy after a period of use of the device, in this case a dressing for a chronic wound. At first glance the two assays—TVC and agar diffusion—could be seen to be mutually contradictory, in that using the TVC assay aged silver alginates have lost the majority of their efficacy after aging while CHX-HMP alginates are still efficacious, although less so than at baseline, whereas using the agar diffusion test there was little difference in performance between the materials. This apparent contradiction can be resolved by considering the two antimicrobial assays used. The agar diffusion model will inherently favour a silver device over a CHX technology, in that the ZOI is in part a reflection of how far across the agar the antimicrobial agent is able to diffuse, and a small ion such as silver will diffuse more readily than the much larger CHX [33]. For this reason, ZOI for silver-based dressings are routinely expected to be greater than for other antimicrobials, and this need not be taken to imply that this will translate into superior clinical performance [33]. Thus the barrier to efficacy in agar diffusion was less, and the reduction in silver owing to the aging did not eliminate all silver, such that a ZOI was still achieved with the residual silver present. The TVCs on the other hand are more equitable in terms of the assessment of the specific antimicrobial, and in this assay the reduction in silver’s efficacy after aging is clear (Table 2).

The 7 day aging step is of course a radical simplification of the clinical environment of such a dressing. Nevertheless, such an assessment is necessary to ascertain whether an influx of wound exudate is likely to reduce the efficacy of the antimicrobial contained within. Alginate dressings are used especially for wounds with high levels of exudate. A fast releasing antimicrobial dressing will provide a large dose of antimicrobial initially, which may have undesired effects; for instance, the most common adverse event associated with the CHX-impregnated vascular access devices described above is contact dermatitis, owing to the high concentration of CHX released from these dressings [11]. Furthermore, if release is not sustained, the antimicrobial will be depleted by diffusion into the wound bed and binding to components of the bacterial cell wall. Our observations suggest that the CHX-HMP alginates had a greater longevity of antimicrobial efficacy than the commercial silver alginate.

## Conclusions

Alginate films with impregnated chlorhexidine hexametaphosphate nanoparticles (CHX-HMP) acted as reservoirs of chlorhexidine (CHX), eluting it into aqueous media in a dose- and time-dependent manner. The elution profile showed an immediate burst release followed by a sustained release over the two weeks monitored. By comparison, an equivalent material containing CHXdg released much less CHX and this was depleted after <24 h. Dressings which display antimicrobial properties for substantially less than the time the dressing is usually left on the wound—commonly 7 days—may provide only shortlived protection and thereafter not offer effective prevention against infection.

CHX-HMP impregnated alginates reduced total viable counts of a range of common wound infecting microorganisms: methicillin-resistant *S. aureus* (MRSA), *P. aeruginosa, E. coli, K. pneumoniae* and *A. baumannii* over 24 h in vitro. The reduction in TVC was less than that of a commercial silver alginate dressing at baseline, but greater than the silver alginate after a period of in vitro aging. The CHX-HMP alginates exhibited a dose-dependent behaviour in an agar diffusion model, with contact inhibition at lower CHX-HMP concentrations (0.05–1%) and remote inhibition at higher concentration (0.5–6%). The ZOI was reduced after aging but was still superior in 4 of the 5 microbes to that of the silver alginate dressing.

While the CHX-HMP alginate is an early prototype, and much optimisation of dose and distribution still remains to be carried out, these data indicate that the material may offer favourable properties for the development of sustained efficacy wound dressings for highly exuding, high infection risk wounds.
